# Exploring the anatomical configurations of the cerebral arteries in a cohort of South African patients

**DOI:** 10.1038/s41598-024-56767-y

**Published:** 2024-03-13

**Authors:** Gugulethu Sibiya, Bukola R. Omotoso, Rohen Harrichandparsad, Lelika Lazarus

**Affiliations:** 1 Discipline of Clinical Anatomy, School of Laboratory Medicine and Medical Sciences, College of Health Sciences, University of KwaZulu-Natal, Westville Campus, Private Bag X54001, Durban, 4000 South Africa; 2 Department of Neurosurgery, Inkosi Albert Luthuli Central Hospital, School of Clinical Medicine, College of Health Sciences, Nelson R. Mandela School of Medicine, University of KwaZulu-Natal, Durban, South Africa

**Keywords:** Anterior cerebral artery, Posterior cerebral artery, Middle cerebral artery, Anatomical variation, Anatomy, Medical research

## Abstract

The cerebral arteries, specifically the anterior cerebral artery (ACA) and posterior cerebral artery (PCA), work together with the smaller calibre arteries to provide effective communication between the anterior and posterior circuits of the brain via the circle of Willis (CoW). Morphologic variations of the cerebral arteries and the CoW may alter blood flow to the brain, resulting in intracranial vascular disorders associated with stroke, and aneurysms. This study aimed to document the morphology of the cerebral arteries and the CoW in the South African population. Two hundred and thirty-nine computed tomography angiography scans were assessed. Cerebral arteries and CoW normal morphology and variations were classified as complete, absent, or hypoplastic. The ACA A_1_ was absent in 4.91%, hypoplastic in 30.40%, fenestrated in 1.06%, and typical in 63.6%. The ACA A_2_ was absent in 0.42%, hypoplastic in 26.28%, and typical in 69.44%. We found triple ACA A_2_ in 2.98%, azygos in 1.28% and fenestrated in 1.28%. The middle cerebral artery (MCA) was hypoplastic in 7.35% and typical in 92.64%. The PCA was hypoplastic in 28.74% and typical in 71.25%. Knowledge of the configuration of the CoW plays a significant role in guiding therapeutic decision-making in treating various neurovascular pathologies.

## Introduction

The cerebral arteries and their branches perfuse the cerebral cortex and, most importantly, form the larger arteries of the circle of Willis (CoW). Specifically, the ACA and PCA work together with the smaller calibre arteries (anterior and posterior communicating arteries) to provide effective communication between the anterior and posterior circuits of the brain. The CoW is a vascular network formed at the base of the skull in the interpeduncular fossa^1^. It is classically described as a symmetrical polygon derived from anastomoses between branches of the internal carotid arteries (ICA) and vertebral arteries (VA)^2^. The anterior cerebral (ACA), posterior cerebral (PCA), anterior communicating (AComA), and posterior communicating arteries (PComA) constitute the CoW. The internal carotid artery also participates in the construction of the CoW. This configuration allows collateral blood flow and pressure equalization^3^. The AComA and PComA are important components of the CoW as they act as collateral channels to stabilize blood flow between the two hemispheres^1^. Adequate collateral circulation across the vessels of CoW will reduce the risk of transient ischemic attack and ischemic stroke. Similarly, a complete CoW has been associated with reduced stroke severity, recurrence, death, and better recovery^4^.

The presence of variation in the configuration of the cerebral arteries that formed the CoW has been associated with cerebrovascular risk factors and intracranial vascular disorders, such as stroke, aneurysms, and white matter hyperintensities^5–7^. The extent of variation of these arteries is not predictable and may include but is not limited to duplication, absence or aplasia, fenestration, hypoplasia, and accessory vessels^1,8,9^. Another rare variation is the azygos ACA. Azygos ACA has been linked with developmental disorders such as holoprosencephaly, neuronal migration anomalies, and aneurysm formation^10^. These morphologic variations may modify the natural, symmetrical geometry of CoW and thus counterbalance the haemodynamics of blood flow, resulting in hypoperfusion in some parts of the brain. Some variations have been identified as risk factors for acute ischemic stroke or related to a neurological disease such as migraine^5,10^. For instance, changes in the diameter of some arterial segments of the CoW due to hypoplasia have been observed in the case of ischemic cerebral accidents^11^. In addition to the associated pathologies, variations have surgical implications. It is important to determine the adequacy of the collateral circulation when planning for procedures to treat intracranial vascular pathologies, which may involve ligations or parent vessel occlusion.

In recent times, most intracranial vascular pathologies have been treated using endovascular procedures, which require knowledge of detailed anatomy and the extent of variation. Prevalence of incomplete or atypical CoW has been reported in various population groups using different image modalities^4,5,12,13^. Previous reports from the South African population used cadaveric samples with a limited sample size^14^. Due to the multiracial composition of the South African population, it is crucial to consider the racial groups to provide detailed information on the configuration of the cerebral arteries and CoW in this region. Racial/ethnic differences associated with the incidence of vascular variation have been identified to contribute partially to the differences in the incidence of some cerebrovascular diseases and cerebral vascular morbidity^1,15^. In the present study, we assessed the morphology and described the variations of the cerebral arteries and the CoW in South African patients using multidetector computed tomography angiography (MDCTA). We also investigated the influence of demographic factors such as age, gender, and race. Such information is essential for diagnosis and treatment using endovascular procedures and surgical interventions.

## Materials and method

### Patient population

This study is a retrospective observational review of 239 MDCTA images of South African patients. The images were obtained from a private hospital in Durban, South Africa, between January 2009 and September 2019. The Biomedical Research Ethics Committee of the University of KwaZulu-Natal approved the study (BREC/00004487/2022) and waived the need for informed consent as this study utilized retrospective chart analyses. All the images were anonymized, with no patient contact or information. All methods were carried out following relevant guidelines and regulations. Exclusion criteria included MDCTA scans that showed no clarity of the cerebral arteries and CoW segments, scans with motion artifacts or poor-quality imaging, and scans performed on foreign patients. The angiographies were from 141 males (59%) and 98 females (41%). The age range of the patients is between 19 and 105 years. The average age of the patients is reported as mean (standard deviation) 65.7 (15.93). Three population groups were included in the present study: Caucasian 139 (58.16%), Indian 66 (27.61%), and Black 34 (14.23%). Race was defined according to the guidelines outlined in the modern systems of racial classification in the Republic of South Africa^16^.

### CTA imaging protocol

The imaging examination was performed on a 64-detector row computed tomography (CT) scanner (Lightspeed CT, GE Healthcare Medical Systems, Milwaukee, WI, USA) with the scanning protocol as follows: 120 kVp, 697 mAs, beam collimation 64 × 0.625 mm, gantry rotation time 0.4 s, section thickness of 0.625 mm, pitch 0.969:1 and reconstruction interval of 0.625 mm. During the procedure, 80 mL of non-ionic iodinated contrast was infused, followed by 40 mL saline, and injected via a double power injector (Medex flowSens, Geubert USA) into the patient’s antecubital vein (4 mL/s)^16^.

### Imaging reconstruction

**Figure 1:**
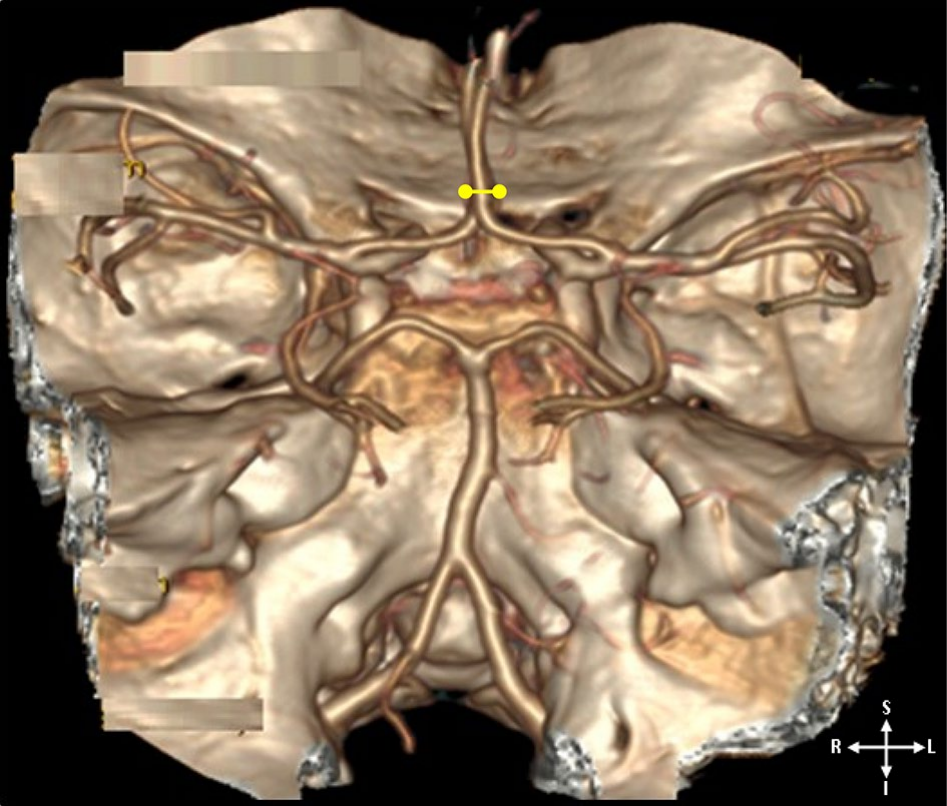
3D-CTA reconstructed image illustrating the intracranial segment of the vertebral arteries, basilar and the internal carotid arteries; typical cerebral arteries dimensions measured for the diameter of each segment shown by the yellow point-to-point measurement.

Postprocessing of three-dimensional images was performed by using a multiplanar reformation (MPR), maximum intensity projection (MIP), multiplanar reconstruction (MPR), and volume rendering (VR) algorithms. The volumetric MDCTA data sets were processed on Advanced Workstation 4.2, (GE Healthcare, Milwaukee, USA)^16^. The CTAs were performed for diagnostic purposes in the context of various cerebrovascular accidents or diseases. Some of the common clinically diagnosed neurological pathologies include transient ischemic attack (TIA), infarction, unilateral body weakness, aneurysm, and ataxia. While some diseases have been linked with vascular variations, the incidence of variation can also be asymptomatic. Therefore, in some cases, the suspected diseases were not found on CT angiography; thus, some materials in this study were derived from a healthy population. Images were analysed using the Picture Archiving Communication System (PACS Syngo Plaza version VB30D https://doclib.siemens-healthineers.com/home). Each radiological image was evaluated for the following parameters, and variables were recorded:

1. The morphology of the following cerebral vessels: the ACA, MCA, and PCA.
2. The ACA, MCA, and PCA diameters were measured using PACS to determine the distance between two points (Fig. 1). Measurements were taken at the maximum part of the segment (the point where the segment was the widest in diameter). Each measurement was taken three times to reduce error, and the average values were used for the statistical analysis.

To be consistent with previous studies^2,17^, vessels with diameters less than one millimeter (1 mm) were classified as hypoplastic (Fig. 4). In the present study, CoW with complete arterial segments were defined as complete CoW (Fig. 1). The accuracy and repeatability of the measurements were determined by random sampling of 25 scans, and a second observer took measurements for inter-observer reliability testing.

### Statistical analysis

Statistical analysis was conducted using SPSS version 28 (SPSS Inc., Chicago, IL, USA https://www.ibm.com/support/pages/ibm-spss-statistics-server280x), and p-values less than 0.05 were considered statistically significant. The distribution of variables was tested using the Kolmogorov–Smirnov test. Because some of the variables are not normally distributed, while some are normally distributed, both parametric and non-parametric tests were used. The Wilcoxon Signed Rank test was used to compare paired samples (gender). The Kruskal–Wallis test was used to determine statistically significant differences in the dependent variables between the three racial groups, and the Chi-square test was used for categorical variables. Some continuous variables are presented as mean (interquartile range), age was presented as mean ± standard deviation, and the categorical variables are represented by a number (N) and percentage. The interclass coefficient correlation (ICC) was used to examine the reliability of measurements.

### Ethics approval

This study was performed in line with the principles of the Declaration of Helsinki. The design was approved by the Institutional Review Board/Ethics Committee (Biomedical Research Ethics Committee of the University of KwaZulu-Natal with ethical No: BREC/00004487/2022).

## Results

### Morphology

**Figure 2:**
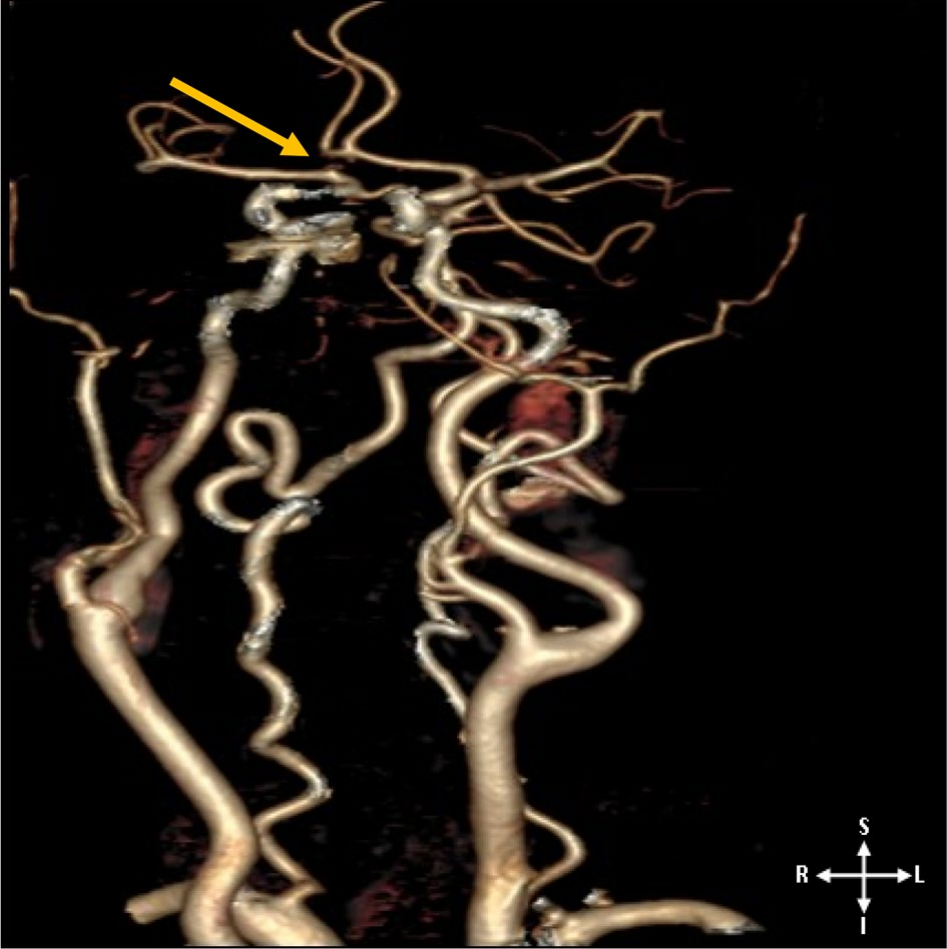
3D-CTA reconstructed image illustrating the anterior and posterior circulation territory; atypical CoW with the absence of a segment (left ACA A_1_).

**Figure 3:**
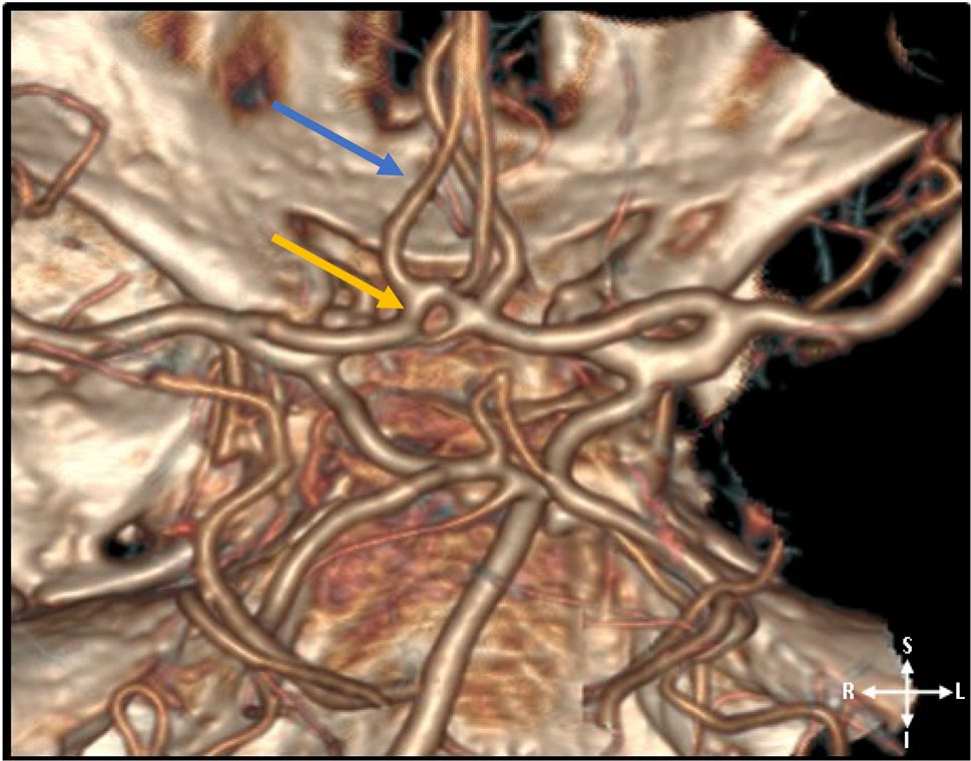
3D-CTA reconstructed image showing the basilar and the internal carotid arteries; atypical CoW. The yellow arrow illustrated a fenestrated left ACA A_1_ segment. The blue arrow illustrated a triple ACA A_2_ segment.

**Figure 4:**
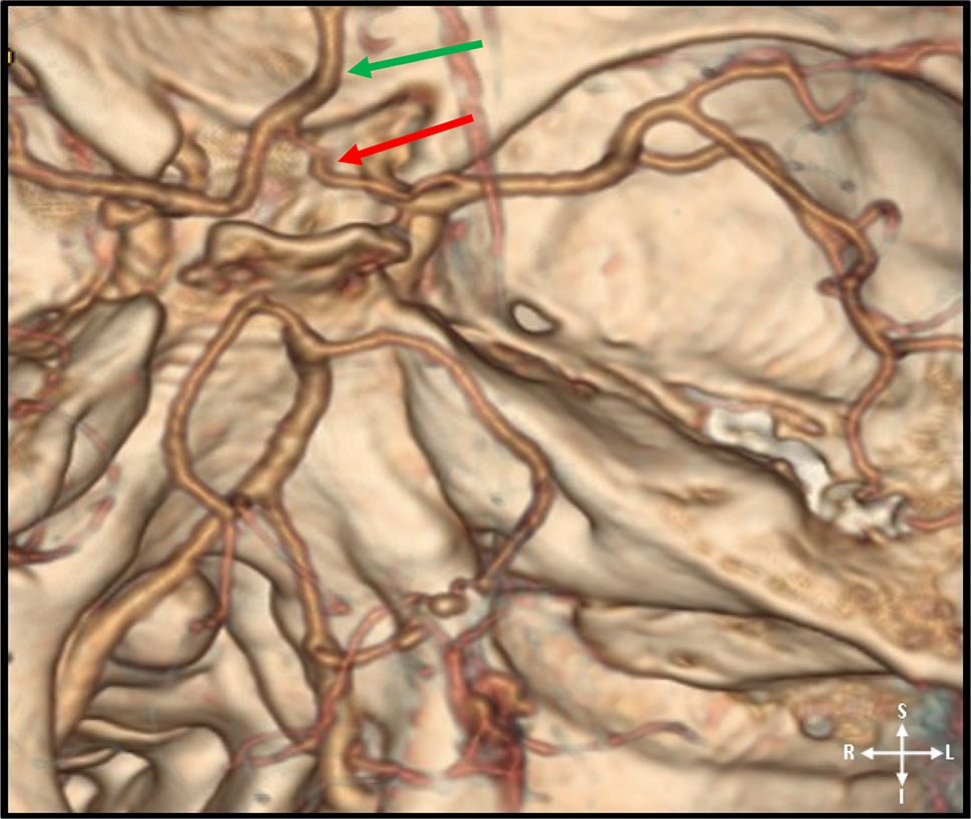
3D-CTA reconstructed image illustrating the the intracranial segment of the vertebral arteries, basilar and the internal carotid arteries and their branches; atypical CoW indicating azygous ACA A_2_ segment (green arrow) and a hypoplastic right ACA A_1_ segment (red arrow).

A complete CoW was seen in 74.23% and variations in 10.84%. The ACA, MCA, and PCA were assessed by laterality for incidence of variations. According to Cilliers et al., the segments of the ACA were described as ACA A_1_ (precommunicating) and ACA A_2_ (postcommunicating)^18^. The most reported variation is hypoplasia affecting the three cerebral arteries. This is followed by absence, triple, fenestration, and azygos ACA, making the ACA the most variable of the three arteries (Figs. 2, 3, 4). Detailed results of the morphology are summarized in Table 1. Regarding the influence of demographic factors on the incidence of variation in the morphology of the cerebral arteries, the parameters of the segments of the arteries were contrasted against age, sex, and race (Table 2, 3, 4). A significant difference was noted in the incidence of variation across the age group on the right involving only ACA A_1_ (Table 2). A partial significant difference between genders was registered involving only the ACA (A_1_ and A_2_ segments) (Table 3). The incidence of the MCA and PCA hypoplasia showed a significant difference across the racial groups (Table 3).

**Table 1:** Incidence of variations in the morphology of the arteries that constituted the CoW diagnosed by MDCTA.

Segment	Variations												Sample size (n)
	Absence (%)		Hypoplasia (%)		Typical (%)		Triple (%)		Fenestration (%)		Azygous (%)		
	R	L	R	L	R	L	R	L	R	L	R	L	
ACA A_1_	7.26	2.56	31.62	29.18	60.25	66.95	–	–	0.85	1.28	–	–	239
ACA A_2_	0.42	–	27.35	25.21	68.37	70.51	1.28	1.70	1.28	1.28	1.28	1.28	239
MCA	–	–	7.98	6.72	92.01	93.27	–	–	–	–	–	–	239
PCA	–	–	27.27	30.21	72.72	69.78	–	–	–	–	–	–	239

*ACA* anterior cerebral artery, *MCA* middle cerebral artery, *PCA* posterior cerebral artery.

**Table 2:** Incidence of variations in the morphology of the arteries that constituted the CoW grouped according to age in South African patients**.**

Age	Segments of CoW and variations																											
	ACA A_1_ (n = 239)								ACA A_2_ (n = 239)												MCA (n = 239)				PCA (n = 239)			
	AB (%)		FE (%)		HY (%)		TY (%)		AB (%)		AZ (%)		FE (%)		HY (%)		TR (%)		TY (%)		HY (%)		TY (%)		HY (%)		TY (%)	
	R	L	R	L	R	L	R	L	R	L	R	L	R	L	R	L	R	L	R	L	R	L	R	L	R	L	R	L
19–29	0	0	50	0	50	50	0	50	0	0	0	0	0	0	50	0	0	0	50	100	50	0	50	100	0	0	100	100
30–39	11.76	5.88	0	0	29.41	29.41	58.82	58.82	0	0	5.9	5.9	0	0	41.2	29.4	5.9	5.9	41.2	52.9	17.65	11.76	82.35	88.24	41.18	35.29	52.94	58.82
40–49	0	0	0	0	50	35	50	65	0	0	5.0	5.0	0	0	25	30.0	0	0	65	60.0	10	10	90	90	40	40	55	60
50–59	4.76	2.38	2.38	0	35.71	21.43	52.38	71.43	0	0	0	0	2.4	2.4	31	28.6	0	0	64.3	66.7	11.90	9.52	88.10	90.47	30.95	35.71	66.67	64.29
60–69	6.82	0	0	2.27	29.55	29.55	59.09	63.64	0	0	0	0	0	0	25	31.8	0	0	75	68.2	13.64	13.64	86.36	86.36	22.73	29.55	70.45	68.18
70–79	10.77	3.08	0	1.54	38.46	35.38	49.23	58.46	0	0	1.5	1.5	1.5	1.5	27.7	18.5	0	0	66.2	75.4	0	1.54	98.46	98.46	27.69	29.23	69.23	67.69
80–89	7.14	2.38	0	2.38	11.90	19.05	80.95	76.19	2.4	2.4	0	0	2.4	2.4	21.4	23.8	2.4	2.4	71.4	69.0	4.76	2.5	95.24	95.24	9.52	19.05	90.48	80.95
90–105	0	14.29	0	0	0	28.57	100	57.14	0	0	0	0	0	0	0	0	14.3	14.3	85.7	85.7	0	0	100	100	42.86	28.57	57.14	71.43
p-value	R: p < 0,001* L: p = 0.921								R: p = 0.052 L: p = 0.581												R: p = 0.154 L: p = 0.430				R: p = 0. 233 L: p = 0.765			

*p-value ≤ 0.05 was considered statistically significant.

*AB* absence, *TY* typical, *HY* hypoplasia, *AZ* azygous, *TR* triple, *FE* fenestration, *R* right, *L* left, *n* sample size.

**Table 3:** Incidence of variations in the morphology of the CoW grouped according to Gender in South African patients.

Gender	Segments of CoW and variations																											
	ACA A_1_ (n = 239)								ACA A_2_ (n = 239)												MCA (n = 239)				PCA (n = 239)			
	AB (%)		FE (%)		HY (%)		TY (%)		AB (%)		AZ (%)		FE (%)		HY (%)		TR (%)		TY (%)		HY (%)		TY (%)		HY (%)		TY (%)	
	R	L	R	L	R	L	R	L	R	L	R	L	R	L	R	L	R	L	R	L	R	L	R	L	R	L	R	L
Male	6.38	2.13	0.71	0.71	39.72	32.62	52.48	63.83	0	0	0.71	0.71	1.42	1.42	31.91	31.91	2.13	2.84	61.70	60.99	9.93	7.09	90.07	92.20	30.50	31.91	66.67	66.67
Female	8.16	3.06	1.02	2.04	18.37	22.45	68.37	67.35	1.02	0	2.04	2.04	1.02	1.02	19.39	14.29	0	0	74.49	80.61	5.10	6.12	93.88	93.88	20.41	26.53	75.51	71.43
p-value	R: p = 0.006* L: p = 0.104								R: p = 0.238 L: p = 0.028*												R: p = 0.199 L: p = 0.672				R: p = 0.208 L: p = 0.641			

*p-value ≤ 0.05 was considered statistically significant.

*AB* absence, *TY* typical, *HY* hypoplasia, *AZ* azygous, *TR* triple, *FE* fenestration, *R* right, *L* left, *n* sample size.

**Table 4:** Incidence of variations in the morphology of the CoW grouped according to Race in South African patients.

Race	Segments of CoW and variations																											
	ACA A_1_ (n = 239)								ACA A_2_ (n = 239)												MCA (n = 239)				PCA (n = 239)			
	AB (%)		FE (%)		HY (%)		TY (%)		AB (%)		AZ (%)		FE (%)		HY (%)		TR (%)		TY (%)		HY (%)		TY (%)		HY (%)		TY (%)	
	R	L	R	L	R	L	R	L	R	L	R	L	R	L	R	L	R	L	R	L	R	L	R	L	R	L	R	L
Black	5.88	0	0	0	41.18	32.35	47.06	61.76	0	0	2.94	2.94	0	0	38.24	38.24	0	0	58.82	58.82	14.71	8.82	85.29	91.18	20.59	41.18	67.65	52.94
Indian	12.12	3.03	0	0	37.88	34.84	48.48	60.61	1.52	0	1.52	1.52	0	1.52	31.82	28.79	0	0	60.61	63.64	16.67	13.64	81.82	84.85	39.39	43.94	59.09	54.55
Caucasian	5.04	2.88	1.44	2.16	25.18	24.46	66.91	68.34	0	0	0.72	0.72	2.16	1.44	21.58	19.42	2.16	2.88	71.94	74.10	2.16	2.88	97.84	97.12	21.58	20.14	76.26	79.14
p-value	R: p = 0.067 L: p = 0.492								R: p = 0.320 L: p = 0.352												R: p = 0.001* L: p = 0.023*				R: p = 0.003* L: p < 0,001*			

*p-value ≤ 0.05 was considered statistically significant.

*AB* absence, *TY* typical, *HY* hypoplasia, *AZ* azygous, *TR* triple, *FE* fenestration, *R* right, *L* left, *n* sample size.

### Diameter

The ICC for intra-observer reliability testing ranged between 86 and 97% for ACA, MCA and PCA. ICC ranged between 84 and 96% for inter-observer reliability testing for ACA, MCA, and PCA.

The average diameter of ACA A_1_ (left-L and right-R), ACA A_2_ (L and R), MCA (L and R), and PCA (L and R) were summarized in Table 5. The average diameter of each of the arteries was contrasted against gender (Table 6), race (Table 7), and age (Table 8) to determine if these demographic factors influenced the size of the arteries. Cerebral arteries with pathological vascular changes such as aneurysms were excluded from the study as this may influence the average diameter values during analysis.

**Table 5:** Average diameter and laterality of the arteries of the CoW.

Parameter	ACA A_1_R, median (IQR)	ACA A_1_L, median (IQR)	ACA A_2_R, median (IQR)	ACA A_2_L, median (IQR)	MCAR, median (IQR)	MCAL, median (IQR)	PCAR, median (IQR)	PCAL, median (IQR)
Diameter (mm)	1.240 (0.85)	1.320 (0.81)	1.320 (0.89)	1.400 (0.88)	2.120 (1.02)	2.165 (1.06)	1.390 (0.88)	1.320 (0.74)
Wilcoxon sign rank p-value	0.130		0.090		0.888		< 0.001*	

*p-value ≤ 0.05 was considered statistically significant.

Results are reported as median (IQR) in mm.

**Table 6:** Average diameter of the segments of CoW in relation to gender.

	Male	Female	Mann Whitney U p-value
ACA A_1_R	1.060 (0.83)	1.350 (0.78)	0.003*
ACA A_1_L	1.320 (0.78)	1.350 (0.82)	0.077
ACA A_2_R	1.320 (0.97)	1.500 (0.79)	0.045*
ACA A_2_L	1.320 (0.91)	1.520 (0.81)	0.009*
MCAR	2.120 (0.97)	2.120 (1.01)	0.317
MCAL	2.165(1.07)	2.160 (0.92)	0.259
PCAR	1.320 (0.96)	1.420 (0.73)	0.243
PCAL	1.240 (0.76)	1.410 (0.69)	0.116

*p-value ≤ 0.05 was considered statistically significant.

Results are reported as median (IQR) in mm.

**Table 7:** Average diameter and laterality of the segments of CoW in South Africa racial groups.

	Black	Indian	Caucasian	Kruskal-Wallis p-value
ACA A_1_R	1.060 (0.80)	1.060 (0.68)	1.320 (0.84)	0.018*
ACA A_1_L	1.180 (1.02)	1.210 (0.84)	1.420 (0.78)	0.271
ACA A_2_R	1.170 (0.84)	1.280 (0.90)	1.410 (0.79)	0.097
ACA A_2_L	1.320 (0.91)	1.320 (0.92)	1.430 (0.81)	0.108
MCAR	1.850 (0.83)	1.850 (1.26)	2.290 (0.89)	< 0.001*
MCAL	1.925 (0.80)	1.760 (1.28)	2.370 (0.84)	< 0.001*
PCAR	1.280 (0.61)	1.130 (0.83)	1.450 (0.83)	0.010*
PCAL	1.060 (0.67)	1.100 (0.85)	1.430 (0.60)	0.001*

*p-value ≤ 0.05 was considered statistically significant.

Results are reported as median (IQR) in mm.

**Table 8:** Average diameters of the segments of CoW in relation to age.

	19–29	30–39	40–49	50–59	60–69	70–79	80–89	90–105	Kruskal–Wallis p-value
ACA A_1_R	0.840 (0.79)	1.060 (0.46)	1.060 (0.53)	1.105 (0.35)	1.320 (0.88)	1.060 (0.80)	1.670 (0.88)	2.020 (1.11)	< 0.001*
ACA A_1_L	1.110 (0.81)	1.210 (0.69)	1.110 (1.04)	1.500 (0.79)	1.335 (1.00)	1.135 (0.73)	1.510 (0.79)	1.510 (1.12)	0.050*
ACA A_2_R	1.070 (0.90)	1.060 (0.59)	1.210 (0.79)	1.320 (0.92)	1.410 (0.86)	1.240 (0.63)	1.630 (0.88)	1.930 (0.36)	0.018*
ACA A_2_L	1.160 (1.06)	1.320 (0.85)	1.240 (1.06)	1.320 (0.92)	1.320 (1.16)	1.380 (0.63)	1.555 (0.99)	2.060 (0.36)	0.061
MCAR	1.895 (0.97)	1.850 (0.95)	1.985 (0.91)	1.935 (1.23)	2.095 0.99)	2.125 (0.83)	2.530 (0.94)	2.050 (1.29)	0.058
MCAL	2.045 (1.18)	1.760 (0.50)	2.030 (0.93)	2.090 (1.12)	2.050 (1.09)	2.080 (0.94)	2.560 (0.85)	2.290 (1.58)	0.010*
PCAR	1.260 (1.20)	1.170 (0.76)	1.320 (0.87)	1.320 (0.77)	1.390 (0.88)	1.320 (0.81)	1.760 (0.78)	1.530 (1.16)	0.002*
PCAL	1.270 (1.13)	1.285 (0.54)	1.105 (0.93)	1.210 (0.76)	1.360 (0.83)	1.210 (0.75)	1.585 (0.98)	1.670 (1.15)	0.031*

*p-value ≤ 0.05 was considered statistically significant.

Results are reported as median (IQR) in mm.

## Discussion

Arteries of the CoW provide collateral circulation and compensation from arteries of the contralateral side in cases of diseases or injury to any of the arteries. Morphologic variations of the cerebral arteries contributing to the CoW have been associated with an increased risk of vasospasm, subarachnoid haemorrhage, and acute ischemic stroke^16^. Authors have reported disparity in the prevalence of these variations across different population groups. In a recent meta-analysis, authors have hypothesized that the prevalence of atypical CoW may be as high as 68%^2^. Also, using human embryo samples, some researchers have reported variation in 85% of the total samples and suggested that variations are congenital and are present from the initiation of CoW formation during embryogenesis^19^. However, other researchers have reported that the complete or classical text-book described variant of the CoW is the most common in their subjects^14,20^. Our findings support the latter report as we found complete CoW in 74% of the patients. Variations such as absence, hypoplasia, duplication, fenestration, accessory vessels, and asymmetry of the arteries have been associated with the alteration of blood flow to the brain tissue and thus enhance vascular diseases^1,7^. For instance, asymmetric CoW configurations have been linked with an increased risk of aneurysm rupture^21^.

The available evidence in the literature from original and review studies using imaging and cadaveric samples has shown that the PcomA hypoplasia/aplasia is the most frequently reported variation of the CoW^2,22,23^. This is followed by the AComA hypoplasia^23^. This situation is responsible for the prevalence of incomplete CoW, as reported by most authors. Despite the non-invasive nature of the CTA and its capacity to display intricate vascular architecture, it has technical limitations in the adequate assessment of arteries with small calibers, such as the communicating arteries. Some authors have hypothesized that CTA may not provide a good image analysis of vessels below 0.6 mm diameter^22^. Thus, most studies that used CTA have reported a high prevalence of absent/hypoplastic/aplasia or invisible PcomA^23,24^. The major role of the communicating arteries is to redistribute blood between adjacent arteries of larger caliber^22^. Therefore, it is sometimes common for the communicating arteries to have smaller diameters, and they have been reported with a high prevalence of hypoplasia/aplasia. This is one of the reasons why a complete CoW is rarely seen on radiographs. Similarly, in the present study, the authors could not investigate each arterial segment of the CoW. The AComA and PComA are not clearly seen in some images and are therefore excluded from the analysis.

The most prevalent variation in the present study is the hypoplasia of the ACA (Table 1). Hypoplasia of the ACA A_1_ segment may significantly disturb perfusion relationships in the AComA area. This may be due to the suspected stress burden at the junction angle between ACA A_1_ and ACA A_2_. This alteration in the blood flow pattern may prompt the formation of an aneurysmal sac^21^. Another rare variation of the ACA is the azygos ACA, in which two ACA A_1_ segments of both hemispheres form a single ACA A_2_ trunk without an AComA^8^. The prevalence of this variation ranges from 1 to 2.3%^7,8,25,26^. The findings from the present study are similar to the previous reports, as we found azygos ACA A_2_ in 1.28% of the patients. Azygos ACA has been identified as a predictor of bilateral frontal strokes and midline central nervous system malformations, such as agenesis of the corpus callosum, holoprosencephaly, and intracranial arteriovenous malformation^27,28^. In contrast, some researchers have suggested that there is no direct evidence of any increased incidence of stroke associated with azygos ACA. However, symptoms resulting from occlusion of azygos ACA can be unique because it can cause ischemic infarction in both cerebral hemispheres and the corpus callosum^28^. Furthermore, saccular aneurysms of the azygos ACA are relatively common, with a prevalence rate between 13 and 71%^27^. The increased susceptibility for aneurysm formation may be attributed to the hemodynamic stress of heightened blood flow in the single azygos ACA, which is usually paired^28^.

The Triplicate A_2_ segment of the ACA, or “triple-A_2_”, is another uncommon anatomical variant suspected to mainly result from the persistence of the embryonic median artery of the corpus callosum^29^. The prevalence of triple-A_2_ in the general population without intracranial pathologies such as aneurysms and infarctions is between 1 and 4%^25,29–31^. Similar to our report, we registered triple-A_2_ in approximately 3% of the patients using MDCTA. On the contrary, Ferre and co-authors, using a similar image modality, reported a prevalence as high as 13%^32^. This may be due to selection bias; according to the authors, approximately 90% of the study population had aneurysms. Triple-A_2_ has been associated with a significantly higher risk of AComA aneurysms^29^. Knowledge of the triple-A_2_ variant in the setting of an AComA aneurysm is critical when planning and executing endovascular and open surgical treatment. If the third A_2_ segment is not anticipated and protected, it may be accidentally occluded during treatment, leading to neurologic complications^29^.

Fenestration is another interesting variation of the ACA commonly encountered in intracranial circulation. This phenomenon is characterized by the division of the lumen of an arterial segment into two distinct endothelial-lined parallel channels, which may or may not share a common adventitia^28^. The prevalence of ACA fenestration has been reported between 0 and 4%^25,33,34^. In agreement with previous reports, we found fenestrated ACA A_1_ and ACA A_2_ segments in 1.06% and 1.28% of the patients, respectively. Fenestrations of intracranial arteries are sometimes asymptomatic; however, they have been linked with a high tendency of developing aneurysms. Histological findings have revealed reduced smooth muscle and collagen fibres at the proximal and distal portions of the fenestrated segments^28^. We also found an absent/aplasia ACA A_1_ (Table 1) segment in 4.91% of the patients. Our report is similar to the prevalence reported in imaging studies 1–6%^33–35^. On the other hand, authors have reported a rate as low as 0.4% using cadaveric samples^25^. ACA A_1_ segment absent/aplasia has been associated with AComA aneurysms^36^. This may be due to compromised blood flow in the asymmetry or incomplete CoW. While some researchers have hypothesized that the frequency of the variation of CoW increases with age, the underlying cause of this relationship is not clear^4,5,11,37^. Hindenes and co-authors have suggested atherosclerosis as a possible cause. However, researchers using human embryo samples have reported incomplete CoW in 17 out of 20 samples, stating that the formation of atypical CoW is congenital^19^. This information suggests that age may not significantly affect the frequency of variation. Similarly, in the present study, age does not significantly affect the frequency of variation in all the segments of the CoW except for the right ACA A_1_ segment (Table 2). The absence of ACA segments and hypoplasia were more prevalent in the older patients (60–105 years), and fenestration was more prevalent in younger patients (19–59 years) (Table 2).

Authors have reported conflicting findings regarding the frequency of variation between males and females. According to some authors, complete CoW is common in females^4,37^, while some specific variations are mostly reported in males or females^38^. Others have reported no correlation between gender and frequency of CoW variation^5,11^. Similar to the present study, there was no statistically significant difference in the prevalence of variation in the morphology of the majority of the CoW arteries in both genders, except for the right ACA A_1_ and left ACA A_2_ segment (Table 3). The discrepancies reported in the above studies may be attributed to differences in sample sizes (predominantly male/female ratio). Differences in the choice of methodology may also make it difficult to compare the results of some morphological variations.

Researchers from different regions have hypothesized racial disparities in the incidence of CoW variation in their population samples. For instance, PCA hypoplasia has been reported across populations at different prevalence rates. Nyasa and co-authors reported an incident rate as high as 13% in the Malawi population^3^ compared to 5% reported in a USA study^39^. Others have reported an even lower rate of less than 5%, including samples from the South African population^14,40,41^. In the present study, hypoplasia of the PCA is higher than 20%, and most of the cases were reported in India, followed by the Black, and the least documented in the Caucasians. Similarly, we reported most of the MCA hypoplasia in India, followed by the Black and a few cases in the Caucasians (Table 4). The significant differences recorded across the racial group in the South African population may be a show of genetic influence since we defined hypoplasia by a similar value of less than 1 mm across the races. However, more data are required to corroborate these findings.

In the present study, the average diameter of the ACA, MCA, and PCA is similar to the values reported by Yeniçeri and co-authors in a Turkish population (ACA A_1_(R-L) 1.24–1.32, ACA A_2_(R-L) 1.32–1.40, MCA(R-L) 2.12–2.16 and PCA(R-L) 1.39–1.32)^12^. A slightly higher value was reported by El-Barhoun and co-authors, using samples from the Australian cohort^13^. Another important finding in the present study is that the diameter of ACA was significantly larger in females than in males (Table 6). Also, the bilateral MCA, PCA, and unilateral ACA A_1_ were significantly larger in the Caucasians compared with the Indians and the Blacks (Table 7). The average diameter seems significantly larger in the older patients for bilateral ACA A_1_ and PCA and unilateral ACA A_2_ and MCA (Table 8). We hypothesised that morphometric parameters such as diameter might vary across gender, race, and age. As most of the previous studies did not compare the average diameter of the cerebral arteries with these demographic factors, it is difficult to compare the findings in the present study with other studies in the literature. More studies with larger sample sizes are required to validate this hypothesis.

### Study limitations

This study investigated variations of cerebral arteries and some arterial segments of the CoW but did not address the communicating arteries because of the imaging modality that was utilized. The AComA and PComA are rarely seen on CTA images due to their smaller sizes and were therefore excluded from this analysis.

## Conclusion

Morphologic variations in the configuration of the cerebral arteries and the CoW are common in the South African population studied in this work. ACA is the most variable of all the arteries; hypoplasia was the most prevalent variation. There was a significant difference in the average diameter across demographic parameters such as gender, race, and age. Further studies are required to establish the morphometric discrepancies associated with the demographic factors, especially in the South African population. Knowledge of the configuration of the cerebral arteries and CoW plays a significant role in guiding therapeutic decision-making in treating various neurovascular pathologies. Awareness of these anatomical variations would be vital in patients' selection for preventative treatment and neurovascular procedures.

## Data Availability

Datasets included in the study are available from the corresponding author upon request.

## Abbreviations

CoW: Circle of Willis
ACA: Anterior cerebral artery
MCA: Middle cerebral artery
PCA: Posterior cerebral artery
